# Functional Age-Related Changes Within the Human Auditory System Studied by Audiometric Examination

**DOI:** 10.3389/fnagi.2019.00026

**Published:** 2019-02-26

**Authors:** Oliver Profant, Milan Jilek, Zbynek Bures, Vaclav Vencovsky, Diana Kucharova, Veronika Svobodova, Jiri Korynta, Josef Syka

**Affiliations:** ^1^Department of Auditory Neuroscience, Institute of Experimental Medicine of the Czech Academy of Sciences, Prague, Czechia; ^2^Department of Otorhinolaryngology of Faculty Hospital Královské Vinohrady and 3rd Faculty of Medicine, Charles University, Prague, Czechia; ^3^Department of Technical Studies, College of Polytechnics, Jihlava, Czechia; ^4^Department of Otorhinolaryngology and Head and Neck Surgery, 1st Faculty of Medicine, Charles University in Prague, University Hospital Motol, Prague, Czechia; ^5^Eye Clinic Liberec, Liberec, Czechia

**Keywords:** presbycusis, central hearing loss, temporal processing, laterogram, cognition

## Abstract

Age related hearing loss (presbycusis) is one of the most common sensory deficits in the aging population. The main subjective ailment in the elderly is the deterioration of speech understanding, especially in a noisy environment, which cannot solely be explained by increased hearing thresholds. The examination methods used in presbycusis are primarily focused on the peripheral pathologies (e.g., hearing sensitivity measured by hearing thresholds), with only a limited capacity to detect the central lesion. In our study, auditory tests focused on central auditory abilities were used in addition to classical examination tests, with the aim to compare auditory abilities between an elderly group (elderly, mean age 70.4 years) and young controls (young, mean age 24.4 years) with clinically normal auditory thresholds, and to clarify the interactions between peripheral and central auditory impairments. Despite the fact that the elderly were selected to show natural age-related deterioration of hearing (auditory thresholds did not exceed 20 dB HL for main speech frequencies) and with clinically normal speech reception thresholds (SRTs), the detailed examination of their auditory functions revealed deteriorated processing of temporal parameters [gap detection threshold (GDT), interaural time difference (ITD) detection] which was partially responsible for the altered perception of distorted speech (speech in babble noise, gated speech). An analysis of interactions between peripheral and central auditory abilities, showed a stronger influence of peripheral function than temporal processing ability on speech perception in silence in the elderly with normal cognitive function. However, in a more natural environment mimicked by the addition of background noise, the role of temporal processing increased rapidly.

## Highlights

– Specific auditory tests reveal the central component of presbycusis.
– Temporal processing deteriorates in the elderly with normal hearing thresholds.
– Speech processing is altered in the elderly without clear cognitive pathology.

## Introduction

The deterioration of hearing with age is a physiological process that starts to become evident after 30 years of age and reaches substantial levels above the age of 60, when it is called presbycusis (Gates and Cooper, 1991). Presbycusis is typically diagnosed in clinical practice by the elevation of hearing thresholds at frequencies above 2 kHz, however the most typical subjective symptom is a deteriorated speech perception under strenuous conditions (background noise; Pronk et al., 2013).

One view on presbycusis is the hypofunction of the auditory periphery. Schuknecht and Gacek (1993) described four types of presbycusis; sensory, metabolic, neural and mechanical, and correlated them with the type of auditory thresholds. Gates et al. (1990) also described four different shapes of auditory thresholds related to presbycusis: sharply sloping, gradually sloping, flat and notched; the proportion of which in the population differs not only according to increasing age but also according to gender.

Another approach to understanding presbycusis was focused on its possible central components as described by the Committee on Hearing, Bioacoustics, and Biomechanics, Commission on Behavioral and Social Sciences and Education, National Research Council (CHABA, 1998). CHABA (1998) and later Humes (1996) and Humes et al. (2012) also took into account speech related difficulties in the elderly, and proposed two additional presbycusis hypotheses: central auditory and cognitive hypotheses. The central auditory hypothesis is based on the additional pathology within the supracochlear parts of the auditory system, which can either result from peripheral pathology or can emerge independently of peripheral pathology. The cognitive hypothesis focuses on the overall effect of aging that leads to cognitive impairment and decreased speech processing (Pichora-Fuller and Singh, 2006; Jayakody et al., 2018). Recently, Füllgrabe and Moore (2014) suggested that the decline in speech perception in older people is partly caused by cognitive and perceptual changes, which is separate from age-related changes in audiometric sensitivity. The most dominant cognitive declines are auditory memory deficits, attention deficits and an executive function that can be correlated with the elevation of auditory thresholds (Pearman et al., 2000; Lin et al., 2011). Willott (1996) in his classical review (1996), differentiated between the central effects of biological aging (connected with the deleterious effects of histopathology and/or pathophysiology of neurons and neural circuits within the central auditory system) and the central effects of peripheral pathology (based on the deleterious effects of the age-related alterations of cochlear neural input to the central auditory system).

One of the most common central pathologies in presbycusis is the inability to detect the temporal features of sound (Grose and Mamo, 2010) which is most likely due to the hypofunction of the inhibitory system responsible for the coding of the rapid sound changes (Suta et al., 2011) or potential neuronal fiber degeneration. Although correlations between the functional/behavioral age-related functional decline and a hearing decline were observed in animal experiments (for review see Syka, 2010), in humans the data is inconclusive. The results of recent clinical studies with presbycusis indicated morphological and biochemical changes in the auditory cortex and auditory pathway (Profant et al., 2013, 2014). Functional changes (Profant et al., 2015; Giroud et al., 2018) in patients with presbycusis show increased activation of the right auditory cortex with aging, which is also supported by the loss of left ear dominance in the periphery (Tadros et al., 2005). However, the degree of hearing loss seemed to have only a minor effect on the changes in the central auditory system (Ouda et al., 2015). Therefore it is unclear whether these changes are due to age related hearing loss, age related cognitive decline or if they solely represent the effect of aging *per se*.

In this study we investigated the auditory functions in elderly participants with clinically normal hearing ability, as detected by auditory thresholds and speech reception threshold (SRT; although these participants exhibited a very mild degree of pathology due to natural aging, their auditory thresholds and SRT did not exceed the clinical normality as defined by WHO), and compared them with young controls. The goal was to improve characterization of age related hearing changes and its specific parameters at different levels of the auditory system in a group of elderly participants with normal hearing by a range of detailed auditory tests. Our battery of tests included examination of the processing of temporal auditory information, its relation to the processing of distorted speech and the possible interactions with cognitive abilities. In clinical practice, elderly patients quite commonly emerge with normal or close to normal hearing thresholds and complain of hearing difficulties related to speech processing, especially in background noise. These deficits cannot be explained by increased hearing thresholds and suggest a central component of the hearing pathology. We hypothesize that even healthy elderly subjects with minimal peripheral pathology and normal cognitive function will display some degree of supracochlear dysfunction, especially in their ability to process temporal parameters of sound.

## Materials and Methods

Fifty-six participants were examined in this study; 28 elderly participants (elderly; 13 women and 15 men) between the ages of 64–79 (mean age 70.4) and 28 young participants (10 men and 18 women) between 17 and 29 (mean age 24.36) were used as controls (young). The elderly participants included in this study had to meet the following criteria: age above 64 years, no subjective hearing loss, auditory thresholds not exceeding 25 dB on speech frequencies. All of the examined participants declined any previous otologic surgery: vestibular lesion, tinnitus, chronic exposure to loud noise, severe head trauma, lesion of the facial nerve, disorder of the cervical spine or had self-reported a central nervous system disorder. None of the participants were musical professionals, but several in the elderly group played musical instruments sporadically. An otoscopic examination, with removal of the cerumen and confirmation of an intact tympanic membrane, was performed on all of the participants. The examination procedures were approved by the Ethics Committee of the University Hospital Motol, in Prague. All participants signed written informed consent.

### Measurement and Stimulation

All acoustic stimuli, except for pure tone audiometry, laterogram and otoacoustic emission (OAE), were presented so that the same signal was delivered simultaneously to both ears. Apart from the measurement of OAEs, acoustic signals were delivered *via* Sennheiser HDA 200 high-frequency audiometric headphones connected to a Madsen Orbiter 922 clinical audiometer. In the case of pure-tone audiometry, the tonal stimuli were generated by the audiometer. In the remaining tests, the signals were either played from a CD or generated by an external generator and routed to the audiometer. The measurements that related to temporal processing employed a custom-made gating device inserted between the signal generator or CD player and the audiometer.

The gating device provided click generation, white noise generation, precise gating of the input signal (used in the gap detection task and the gated speech task), and also a mutual temporal shift of the two auditory channels (used in the laterogram task). The gating device is equipped with two separate audio channels controlled by a PIC18F4550 controller and operated *via* USB from a standard PC using custom-made software. Each channel is provided with an attenuator (1 dB step) and variable rise/fall gating with a variable time shift between channels. The parameters can either be set manually or dedicated software modules can be used for the specific measurement tasks (e.g., the laterogram, see below).

The audio equipment was calibrated using the Brüel and Kjær 2231 sound level meter and Brüel and Kjær 4153 Artificial Ear equipped with Brüel and Kjær 4134 pressure microphone. The pure tones were calibrated according to ISO 389-5, ISO 389-8. Clicks were measured as peak-to-peak equivalent SPL according to IEC 60645-3. The speech level was measured as C-weighted equivalent SPL according to IEC 60645-2. The OAEs were measured using the Otodynamics ILO 292 analyzer and calibrated using the 2 ccm cavity (coupler) supplied by the manufacturer.

#### Pure Tone Audiometry

Pure tone audiometry was measured over an extended frequency range from 125 Hz to 16 kHz (specifically, at 0.125, 0.25, 0.5, 0.71, 1, 1.6, 2, 3.15, 4, 6.3, 8, 10, 12.5, and 16 kHz). Hearing thresholds were measured separately for each ear with a resolution of 2 dB. No significant differences between the left and right audiograms were found (see “Results” section), therefore the thresholds of both ears were analyzed together. Pure tone average (PTAV) was calculated as an average hearing loss at 0.5, 1, and 2 kHz. Hearing loss was also expressed in % according to Fowler correction (Fowler and Sabine, 1942) that uses an average value related to the degree of hearing loss at 0.5, 1, 2 and 4 kHz (the weighted value for each frequency and level of hearing loss is estimated on the basis of the importance of each frequency within the auditory field for speech perception). To express the hearing thresholds over the entire frequency range in a single number, a weighted average of the hearing threshold levels was calculated. Weighting coefficients were estimated with respect to speech recognition capability (highest weight at 2 kHz) with high frequency preference, such as a counterbalance to PTAV, which does not even take into account frequencies above 2 kHz. Coefficients were set with an average equal to 1 [example: if hearing loss is equal for all frequencies, e.g., 10 dB HL, then the result is that value (i.e., 10 dB HL)]. Coefficients for frequencies between 1 and 8 kHz were greater than 1, for frequencies outside of this range were less than 1 and coefficients for frequencies below 500 Hz were even lower. For a typical audiogram of an elderly participant, which has a loss of about 10 dB up to 2 kHz, a slight decrease up to higher frequencies and a sharp drop above 6 kHz (high frequency slope), the result is about 24 dB. The goal to use pure tone audiometry, was to gain more detailed information about the volunteers hearing within the extended frequency range in comparison with standard clinical practice (testing up to 8 kHz).

#### Speech Audiometry

For speech audiometry in silence, a standard CD recording of Czech word audiometry, according to (Seeman, 1960), was used. One set of ten words was presented at each intensity and the recognition score (percentage of understood words) was determined. The measurement started at 60 dB SPL; should the recognition score at this intensity be lower than 70%, the intensity was increased to 80 dB SPL. Subsequently the sound level was decreased with 10 dB steps until near-zero intelligibility was reached. The threshold was stated as the intensity where the recognition score equaled 50%. Speech audiometry is a basic clinical test. The goal was to use clinical information from the test and compare its contribution with the results of more detailed speech tests.

#### Speech in Noise Audiometry

For the speech audiometry in noise, a standard CD recording of Czech sentence audiometry, according to (Dlouhá et al., 2013), was used. The speech level was kept at 65 dB SPL, while the background babble noise level was increased from 64 to 76 dB in 2 dB steps. Ten sentences were presented for each noise level and the recognition score (percentage of sentences understood) was recorded. A correctly understood complete sentence was counted as 1; a partially understood sentence was counted as half. A psychometric function was constructed by plotting the recognition score as the function of the noise level; the noise level at which the psychometric function crossed 50% was taken as the result. Speech audiometry in noise provides improved information on speech processing compared to basic speech audiometry, and mimics the life situations that cause hearing problems in the elderly.

#### Periodically Gated Speech (Chopper)

Short sentences (unused sentences from the set created for the speech audiometry in noise) were periodically gated (cycle duration 200 ms) with a given duty cycle (approx. 30% to 70%); the percentage represents the proportion of the total cycle duration containing the speech signal, the remaining segment of the cycle was muted. The signal was ramped using a raised cosine ramp with 15 ms duration. Gated sentences were generated using a CD player and a custom-made gating device. Ten sentences were presented for each value of duty cycle and the recognition score (percentage of sentences understood) was recorded. The measurement proceeded from small duty cycles (usually 20%) to larger duty cycles in 10% steps until the recognition score reached nearly 100%. It should be noted that the ascending order of duty cycles provides different results than the descending order, hence it was important to strictly adhere to the procedure. This is due to the fact that the person learns during the test. In the case of an ascending order, when the person at first does not understand, his/her chance to learn is restricted. A correctly understood complete sentence was counted as 1; a partially understood sentence was counted as half. The recognition score was plotted as the function of the duty cycle, and the duty cycle corresponding to the 50% recognition score was taken as the result. Chopper is the most complicated speech test in our battery of tests and focuses on central speech processing and its integration with cognitive abilities (specifically working memory, auditory memory and attention deficits).

#### Binaural Time-Intensity Interchange Ratio (Laterogram)

This measurement is employed to evaluate the ability to perceive interaural time (ITD) and interaural level differences (ILD) that the auditory system uses for space perception: when a sound source is deviated from the medial plane, the sound at the contralateral ear has a lower intensity than in the ipsilateral ear due to an acoustic shade of the head, and it arrives with a certain delay due to the longer path. Participants were exposed to stimuli with a different interaural time and intensity difference. The stimuli consisted of trains of 10 clicks (100 μs duration; 100 ms repetition rate; SPL = 100 dB, measured as peak-to-peak equivalent SPL). Each click pair had a certain ITD (−500 μs to 500 μs, 50 μs step) and ILD (−15 dB to 15 dB, 1 dB step) between the left and right ear. The time-intensity trading ratio allows us to compensate one parameter (simulating lateralization to one side) by another parameter (simulating lateralization to the other side), resulting in the perception of the signal in the middle position. Other combinations lead to a lateralized perception. The participants had to indicate their subjective perception of sound source lateralization (left; right; center). The laterogram test was developed to evaluate the integration of bilateral auditory information, providing the possibility to separately evaluate its temporal and intensity aspects. Based on the physiological processing of the auditory signal, it enables the evaluation of the processing of acoustical signals at the subcortical levels.

#### Detection Threshold of Gap in Noise (Gap)

For the gap detection measurement, trains of three successive pauses in a continuous white noise at 70 dB SPL (150 ms intervals between gaps) were presented randomly in time. The subject had to indicate by pressing a button whenever he/she perceived the gaps. Starting at 10 ms, the gap duration was varied (2 ms up, 1 ms down) until the approximate detection threshold was obtained. Close to the approximated threshold, a fine threshold was subsequently determined by varying the gap duration with 0.1 ms steps in a zig-zag manner. The resulting value of the threshold was the gap duration for which the subject’s detection scores equaled 50%, i.e., the duration at which the subject correctly responded in 50% of trials. Gap is widely used as the test for evaluating temporal processing abilities. Based on the results of animal experiments it is believed that it takes place in the auditory cortex. The comparison of gap (cortex) and ITD (subcortical level—most probably inferior colliculus) results enables the differentiation between the two levels of auditory temporal processing.

#### Detection Threshold of Clicks

The auditory thresholds were also measured for clicks, with the aim to exclude frequency specificity and to take into account the processing of extremely short stimulus durations. The detection threshold of short clicks, depending on their level, was measured analogously to the measurement of detection thresholds of pure tones. Trains of three rectangular pulses (100 μs duration, 100 ms inter-pulse interval) were presented to both ears. The intensity of the click trains was varied until the detection threshold was obtained. The initial intensity was estimated at approximately 20 dB above the expected threshold. The intensity of the click trains was varied until the detection threshold was obtained. The level of the click in dB was defined as a peak-to-peak equivalent value. The click threshold was used to minimize the effect of the frequency specific hearing pathology (high frequency hearing loss in elderly) on the cochlea.

#### Detection of Short Tones

The hearing thresholds for tone at 1 kHz were measured as the function of tone duration. The procedure for obtaining the threshold was analogous to the measurement of the pure-tone hearing threshold however, for detection of short tones the signal was presented to both ears and the resolution was improved to 1 dB. The hearing threshold for tone durations of 70, 30, 20, 10, and 5 ms were measured. The short tones examination was used with the aim to exclude the high frequency hearing loss (there were no pathologies at the level of 1 kHz in the elderly group) and its integration with temporal processing (threshold for different duration). Stimulus duration coding is another temporal feature that, based on the data from animal experiments, occurs at the level of cochlea and cochlear nerve.

#### OAE Measurement

The distortion product OAE (DPOAE) and the transiently evoked OAE (TEOAE) with and without contralateral white noise of 70 dB SPL were recorded in both ears. The difference between the response with and without the noise was considered as the suppression. The stimulus for the TEOAEs was the conventional, broadband nonlinear click (0.5–6.0 kHz), with stimulus gain adjusted to 81 dB peak. The stimuli for eliciting DPOAEs at 2f_1_-f_2_ were two primary tones (f_2_/f_1_ = 1.22 with L_1_ = 70 and L_2_ = 70 dB SPL). Both TEOAE and DPOAE provide information about the function of the outer hair cell population. The addition of contralateral suppression allows the evaluation of the function of the efferent auditory pathway.

#### MoCA

Montreal Cognitive Assessment (MoCA; Nasreddine et al., 2005) was used for the assessment of cognitive abilities of examinees. Three versions of Czech variant (Reban, 2006) were used with a maximum score of 30, and the borderline for cognitive impairment set at 26 (Rektorová, 2011).

### Data Analysis

#### Laterogram Analysis

**Figure 1:**
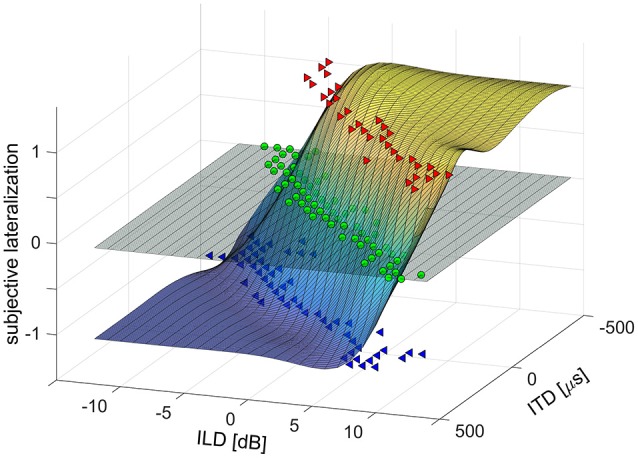
Example of a laterogram plot in 3D space [interaural time difference (ITD) × interaural level difference (ILD) × response value] evaluated by means of non-linear surface fitting.

For the analysis of the laterogram, the responses were plotted in a 3D space (ITD × ILD × response value) and evaluated by means of non-linear surface fitting. Lateralization responses were assigned numerical values (−1 = left, 0 = center, 1 = right) to create a real function of two variables, ITD and ILD. This function was interpolated using a 2D smoothing cubic spline interpolation, to obtain a smooth and high-resolution approximation of the subject’s lateralization responses over the whole range of ITDs and ILDs (see Figure 1). First, to determine the subject’s ability to trade off between ITD and ILD, the interpolated surface was cut with a horizontal plane at zero (i.e., at the “center” response, see Figure 1). The obtained curve was fitted with a third order polynomial and the coefficient of the linear term, determining the slope near the origin, was evaluated. Second, to separate the contribution of ITD and ILD parameters alone, the interpolated surface was cut with two vertical planes, one at ILD = 0, the other at ITD = 0. The resulting curves were subsequently fitted using a sigmoidal Boltzmann-like function to obtain a parameterized approximation of a psychometric function. The sensitivity to ITD or ILD was evaluated as the slope (first order derivative) of the fit in the mid-point. The sensitivity to ITD and ILD may be interpreted as an index of temporal and intensity resolution of the auditory system.

#### The Analysis of Detection of Short Tones

The analysis of short tone detection utilized the fact that the thresholds increase with shorter durations; the shape and steepness of this dependence was quantified using an exponential fit. The worsening of the threshold relative to the threshold for the 70 ms tone was plotted as the function of the tone duration, obtaining a decreasing curve converging to zero. The function was fitted with a single exponential curve in the form of *A*exp(-*kt*) and both parameters *A* and *k* were taken as the results. This approach allowed us to quantify the steepness of the dependence between the tone duration and its detection threshold independently of the absolute detection thresholds.

#### Statistical Analysis

Normality of distributions of the data sets was tested using the Lilliefors test prior to subsequent analyses. Some data sets were found to have a distribution significantly deviating from the normality. For this reason, two-sided Wilcoxon rank sum tests were computed for comparisons of the medians of two data sets. For easier interpretation of the results however, the data are presented as mean ± SEM rather than medians. The possible relationships between the selected parameters were tested using Spearman’s correlation coefficient. In all cases, the alpha level was set to 0.05. Statistical analyses were performed using Matlab software (Mathworks, Inc., Natick, MA, USA).

## Results

### Hearing Thresholds

**Figure 2:**
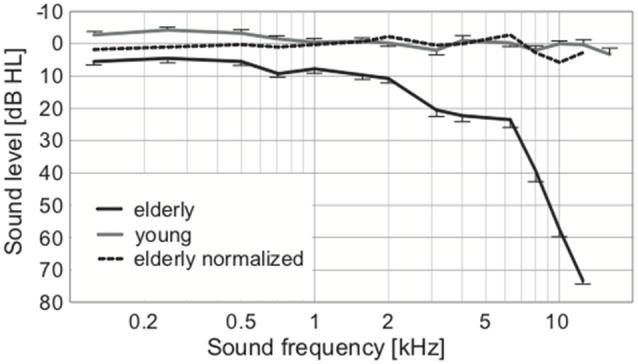
Average audiograms of elderly and young, with the broken line expressing the normalized values of elderly according to correction from Jilek et al. (2014) suggesting physiologic auditory thresholds in our elderly group.

**Figure 3:**
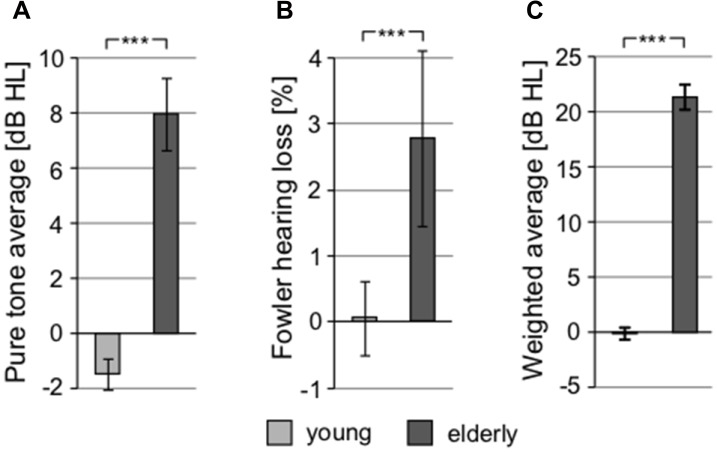
All graphs show the comparison of different threshold-related values used in audiometry, showing significant differences between young and elderly. **(A)** Comparison of pure tone average (PTAV; 0.5, 1, 2 kHz). **(B)** Comparison of hearing loss according to Fowler correction. **(C)** Comparison of weighted averaged thresholds computed over the whole frequency range (***p < 0.001).

To exclude the possibility of biased results due to asymmetry of hearing thresholds, the differences between the left and right hearing thresholds at individual frequencies in all participants were calculated. The difference was not significant (0.11 ± 0.29 dB HL, *p* = 0.25, Wilcoxon sign rank test). The auditory thresholds of the elderly volunteers were elevated compared to the young, particularly above ca. 2 kHz (Figure 2). When PTAV was used to express the hearing function (Figure 3A), the differences between young and elderly values were statistically significant (7.97 ± 1.06 vs. −1.46 ± 0.43 in dB HL; *p* < 0.0001, Wilcoxon rank sum test), however from a clinical point of view such a difference is considered non-significant. Similar differences were present when expressed using Fowler weighting coefficients (2.78 ± 0.6 vs. 0.07 ± 0.05 in %; *p* < 0.0001, Wilcoxon rank sum test; Figure 3B). The most profound differences in expressing hearing thresholds as a single number were present when a weighted coefficient for each measured frequency up to 16 kHz was used (23.44 ± 1.43 vs. −0.13 ± 0.55 in dB HL; *p* < 0.0001, Wilcoxon rank sum test; Figure 3C).

**Figure 4:**
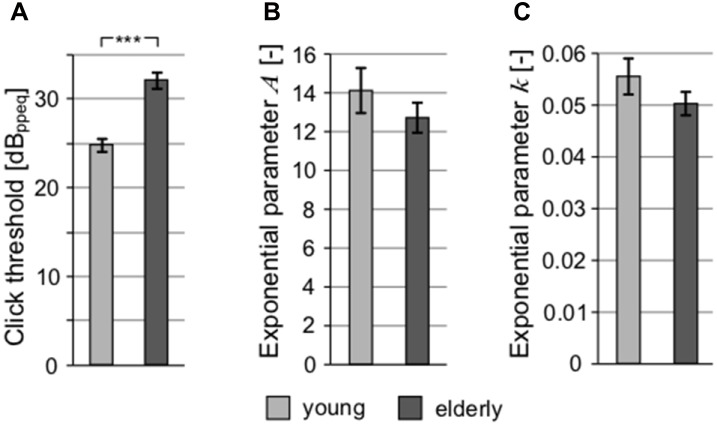
All graphs show the comparison of different threshold-related values. **(A)** Comparison of click threshold. Comparison of parameters of exponential fits of thresholds for 1 kHz tones depending on their duration expressed as exponential parameter A **(B)** and exponential parameter k (**C**; ***p < 0.001).

The auditory thresholds were also measured for clicks (with the aim to exclude frequency specificity and take into account the processing of extreme short stimulus durations; Figure 4A) and short tones at 1 kHz (Figure 4B). These types of stimuli might also reveal a possible auditory neuropathy (Zeng et al., 2005). Although the mean click threshold values in both groups (young: 24.89 ± 0.81 dB_ppeq_; elderly: 32.17 ± 0.85 dB_ppeq_) were higher compared to mean PTAV due to different measuring techniques and units (PTAV is expressed in dB HL, click level as dB peak-to-peak equivalent), the age-related elevation of hearing thresholds measured using clicks is similar as in the case of PTAV (click: 7.29 dB vs. PTAV: 9.43 dB), indicating that both quantities convey similar information about the state of hearing. Therefore, despite its simplicity, the PTAV correlated strongly with the weighted average of hearing loss across the whole frequency range (*ρ* = 0.71, *p* < 0.0001, Spearman correlation), with the Fowler’s coefficient (*ρ* = 0.75, *p* < 0.0001, Spearman correlation), and with the thresholds for click (ρ = 0.69, *p* < 0.0001, Spearman correlation). Therefore it seems that in the case of the normal aging process, the PTAV conveys very similar information as the other measures of hearing thresholds. For this reason, we chose the PTAV for further joint analyses. In the case of 1 kHz threshold dependent on the tone duration, the difference between the groups was not significant for any of the exponential parameters (parameter *A*, young: 14.12 ± 1.16 vs. elderly: 12.72 ± 0.77; parameter *k*, young: 0.055 ± 0.0035 vs. elderly: 0.050 ± 0.0022; *p* > 0.2 in all cases, Wilcoxon rank sum tests), suggesting minimal age related pathology (Figures 4B,C).

### Otoacoustic Emissions

The function of the outer hair cells was examined by recording TEOAE and DPOAE. Overall, the presence of measurable TE was 100% in young vs. 76% in elderly and in the case of DP was again 100% in young vs. 92% in elderly. Both types of OAE showed significant differences in their respective amplitudes, with lower values in elderly (TEOAE, young: 11.63 ± 1.13 dB vs. elderly: 3.58 ± 1.51 dB SPL, *p* < 0.001, Wilcoxon rank sum test; DPOAE, young: 12.26 ± 0.7 dB SPL vs. elderly: 3.1 ± 0.91 dB SPL, *p* < 0.0001, Wilcoxon rank sum test). The contralateral suppression of OAEs showed the effectiveness of the efferent auditory pathway. In both groups the decrease of amplitude caused by the contralateral stimulation, was similar for TE (young: 1.68 ± 0.19 dB SPL vs. elderly: 1.91 ± 0.27 dB SPL, *p* = 0.78, Wilcoxon rank sum test) and also for DP (young: 0.87 ± 0.24 dB SPL vs. elderly: 0.42 ± 0.23 dB SPL, *p* = 0.06, Wilcoxon rank sum test).

### Speech Perception

**Figure 5:**
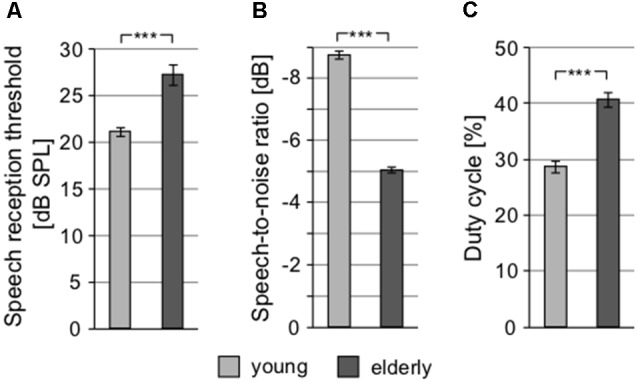
Speech perception tests show significant differences between young and elderly. **(A)** Speech reception thresholds (SRTs). **(B)** Speech-to-noise ratio at which participants reach 50% recognition score (negative SNR values mean higher noise level). **(C)** Proportion of signal in the gated speech needed for 50% recognition score (***p < 0.001).

To examine speech perception, different tests were used. SRT, quantifying the ability to understand speech in silence, showed significantly different values in elderly and young, yet again not exceeding clinically normal limits in either group (young: 21.14 ± 0.45 dB SPL vs. elderly: 27.28 ± 1.12 dB SPL; *p* < 0.0001, Wilcoxon rank sum test; clinically normal limits for SRT are set at 30 dB SPL; Figure 5A). In addition, the difference between the groups (6.14 dB) was similar to the differences in PTAV and click detection threshold (see above), suggesting that despite different absolute values, these three measures quantify the hearing impairment in a very similar way and probably provide the same information about the state of the hearing system. In the speech in babble noise test (SIN), the level of background babble noise resulting in a 50% recognition score was 73.74 ± 0.52 dB SPL in young, compared to 70.68 ± 0.32 dB SPL in elderly (*p* < 0.0001, Wilcoxon rank sum test; Figure 5B). For further testing of speech perception and cognitive abilities of our participants, we employed the so-called chopper test based on the ability to understand periodically gated speech. The results of chopper test again displayed significant differences between young and elderly; the lowest proportion of speech signal needed to reach a 50% recognition score was in young 28.71 ± 1.00% and in elderly 40.85 ± 1.28% (*p* < 0.0001, Wilcoxon rank sum test; Figure 5C). However, the results of the chopper measurements should be treated with caution as they are yet to be validated. As a more complex indicator, the Gardner-Robertson classification scale (Gardner and Robertson, 1988) was used to integrate speech perception and auditory threshold with the aim to use a clinical assessment of serviceable (grade 1, 2)/non-serviceable (grade 3, 4 and 5) hearing, showing only grade 1 (PTAV ≥ 30 dB HL and ≥70% speech discrimination score at dB SPL stimulus) in both groups.

### Binaural Interactions

**Figure 6:**
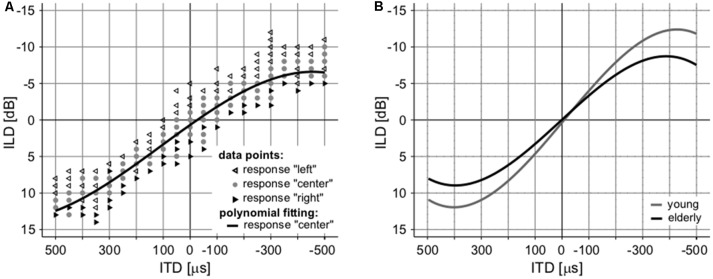
An example of a laterogram of one subject. **(A)** Averaged laterograms of young controls and elderly group **(B)** showing a shallower slope of the trading function in the elderly.

**Figure 7:**
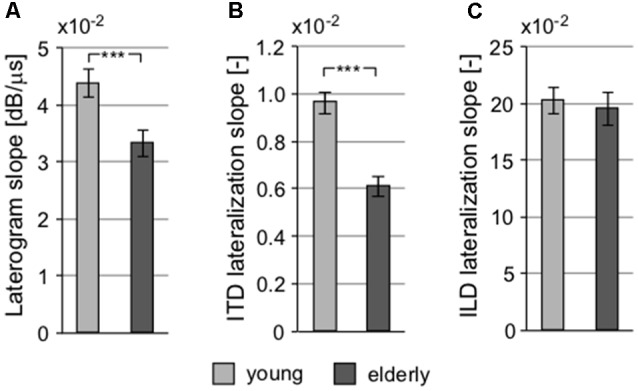
Several parameters of the laterogram suggest significant differences in extracting binaural auditory information at subcortical levels. **(A)** Slope of the laterogram compares the ability to trade between ITD and ILD and is significantly shallower in the elderly. This is probably due to the reduced ability to process the temporal differences between the auditory inputs indicated by the different ITD lateralization slope **(B)** without any pathology in the processing of the intensity parameter, the ILD lateralization slope (**C**; ***p < 0.001).

The interaction of intensity and temporal parameters of sound within the bilateral auditory pathways was examined by the so-called laterogram. Figure 6 depicts an example of one laterogram measurement, along with averaged laterogram curves. The collected data revealed significant differences between both groups in their ability to trade between ITD and ILD as demonstrated by the laterogram slope (young: 0.044 ± 0.002 dB/ms vs. elderly: 0.033 ± 0.003 dB/ms; *p* < 0.001, Wilcoxon rank sum test; Figures 6B, 7A). A shallower slope found in the elderly group indicates that a larger ITD is needed to compensate for a given ILD in the elderly. However, as the laterogram slope depends on two parameters, this result alone could be caused by age-related changes in the detection of both ITD and/or ILD. For this reason, we also analyzed the subjective sound source lateralization for the ITD and ILD separately. We found that while the ILD-induced lateralization was similar in both groups (ILD lateralization slope, young: 0.203 ± 0.012 vs. elderly: 0.196 ± 0.015; *p* > 0.55, Wilcoxon rank sum test; see Figure 7C), the ITD-induced lateralization was significantly smaller in the elderly (ITD lateralization slope, young: 0.0097 ± 0.0005 vs. elderly: 0.0062 ± 0.0004; *p* < 0.0001, Wilcoxon rank sum test; Figure 7B). Hence the different laterogram slope was mainly due to lower sensitivity to the temporal parameter of the signal. Similar as in the case of chopper, data from laterogram measurements should be treated with caution as they are yet to be validated.

### Temporal Parameters

**Figure 8:**
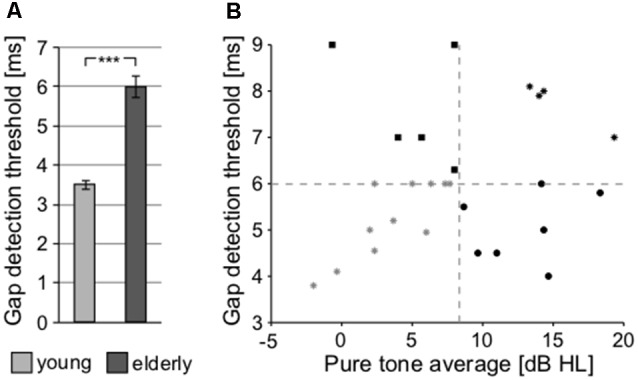
**(A)** Processing of the temporal parameter of sound (gap in noise detection threshold) at cortical level (***p < 0.001). **(B)** Relationship between the gap detection threshold (GDT) and PTAV values for individual participants of the elderly group, providing an option for the further characterization of presbycusis subpopulations.

To further investigate the processing of the temporal parameters of the sound, a gap detection threshold (GDT) test was used (Figure 8A). Significant differences between both groups were observed (young: 3.504 ± 0.114 ms vs. elderly: 6.007 ± 0.278 ms; *p* < 0.0001, Wilcoxon rank sum test), confirming the finding that the elderly participants exhibit poorer temporal processing, as already observed with the ITD-based lateralization. Gap detection is processed at the level of the temporal cortex (Rybalko et al., 2010; Mitsudo et al., 2014) and thus it was chosen for further analyses as a fundamental measure of the central auditory processing, along with the PTAV, as a fundamental index of peripheral processing. When the GDT and PTAV are plotted together in a 2D space (Figure 8B), it is clear that they are only unsubstantially related (*r* = 0.14, *p* = 0.41, Spearman correlation) and that besides people with both parameters, either good or bad, there also exist participants with physiological peripheral processing and deteriorated central processing (top-left corner) or participants with physiological central processing and deteriorated peripheral processing (bottom-right corner). The combined analysis of PTAV and GDT appears to be a promising method for a more detailed characterization of presbycustic subpopulations.

### Cognitive Abilities

The cognitive abilities of volunteers from both groups were assessed by a MoCA questionnaire. The average scores were 28.79 ± 0.29 in young and 27.06 ± 0.44 in elderly (*p* < 0.01, Wilcoxon rank sum test), the lowest score in the young was 26 and in elderly was 23 (three participants scored lower then 26 within the elderly).

### Correlation of Peripheral and Central Auditory and Cognitive Parameters

**Figure 9:**
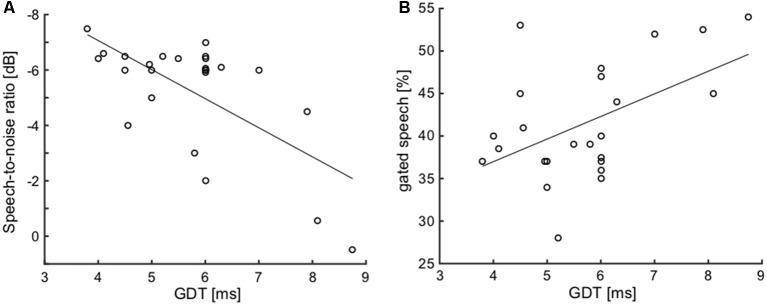
Correlations of GDT with SIN **(A)** and with gated speech **(B)** suggest the importance of the temporal processing factor for speech understanding in a complex listening environment.

To examine the possible relationships between various parameters of interest, joint analyses of selected parameters were performed in the elderly group. First, we focused on the correlation of hearing thresholds with parameters of the function of central auditory system. Considering the temporal parameters, the PTAV (as well as the other peripheral measures) was not correlated with either of them (ITD lateralization slope: ρ = −0.29, *p* > 0.05; GDT: ρ = 0.27, *p* > 0.05; Spearman correlation). However, the speech comprehension measures, especially the SRT, were all correlated with the PTAV (SRT: ρ = 0.75, *p* < 0.0001; SIN: ρ = −0.58, *p* < 0.01; gated speech: ρ = 0.53, *p* < 0.01; Spearman correlation). Hence it appears that the hearing threshold is a strong predictor of speech comprehension ability. The GDT was found to be weakly correlated with the ITD lateralization slope (ρ = −0.37, *p* = 0.05, Spearman correlation), the correlation is nevertheless at the edge of statistical significance and therefore these two temporal measures could most likely be taken as independent. The GDT was also not correlated with the speech recognition threshold (ρ = 0.32, *p* > 0.05, Spearman correlation). On the other hand, the GDT significantly correlated with the ability to understand distorted speech, especially the speech in babble noise (SIN: ρ = 0.51, *p* < 0.01; gated speech: ρ = 0.41, *p* < 0.05; Spearman correlation; Figures 9A,B).

From the non-auditory parameters, we focused on the MoCA index and age. Interestingly, the MoCA index was not related to speech comprehension ability (in all three cases ρ < 0.35, *p* > 0.05, Spearman correlation). It was also not correlated with GDT (ρ = −0.15, *p* > 0.05, Spearman correlation), ITD slope (ρ = 0.27, *p* > 0.05, Spearman correlation), and age (ρ = −0.32, *p* > 0.05, Spearman correlation). However, the MoCA depended on PTAV—the larger the hearing loss, the lower the MoCA index (ρ = −0.57, *p* < 0.01, Spearman correlation). In the case of age within the elderly group, only the ITD slope changes significantly (ρ = −0.53, *p* < 0.01, Spearman correlation); all the other tested parameters (PTAV, SRT, SIN, gated speech, GDT) do not depend on age within the elderly (in all cases ρ < 0.25, *p* > 0.05, Spearman correlation). Considering that these parameters all differ between the elderly and young, it can be assumed that age-related changes do occur, but either more slowly than can be displayed by the variability of age within the elderly, or that the progress of changes with age is not linear or gradual.

The correlation takes into account all the measured data points. Inspired by the plot shown in Figure 8B, it was interesting that the group of participants have deficits in central processing, despite having relatively good periphery. In the plot, these participants appear in the top-left quadrant. Specifically, we extracted the elderly participants who have PTAV lower or equal than median, and GDT higher or equal than median (GDT impaired). We subsequently compared these participants with participants from the top-right quadrant (similar GDT, but worse hearing thresholds-worst hearing), and bottom-left quadrant (similar thresholds, but better GDT-best hearing).

**Figure 10:**
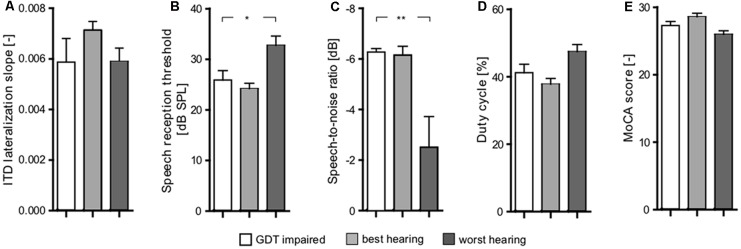
Comparison of three groups of elderly subjects (GDT impaired, best hearing, worst hearing) based on their peripheral (PTAV) and central (GDT) functions inspired by Figure 8B; shows significant differences between worst hearing and both other groups in SIN (*p* < 0.01; **C)** and SRT (*p* < 0.05) parameters **(B)** and close to statistical significance [Cliff’s *d* was medium-to-large (*d* = 0.62)] in gated speech **(D)**. In none of the other parameters [ITD lateralization slope **(A)** Montreal Cognitive Assessment (MoCA) **(E)**] was a significant difference present. Comparison of best hearing and GDT impaired did not show any significant differences (*p < 0.05, **p < 0.01).

The examined parameters were ITD slope, SRT, SIN, gated speech, and MoCA (Figure 10). Interestingly, the GDT impaired participants exhibited no significant differences (*p* > 0.05 in all cases, Wilcoxon rank sum tests) of the tested parameters in comparison with the group of the worst hearing participants (bottom-left quadrant). When compared with the worst hearing participants (top-right quadrant), these GDT impaired participants have better SRT (GDT impaired: 25.9 ± 1.9 dB SPL vs. worst hearing: 32.75 ± 1.8 dB SPL, *p* < 0.05; Wilcoxon rank sum test; Figure 10B) and SIN (GDT impaired: −6.27 ± 0.14 dB vs. worst hearing: −2.51 ± 1.2 dB, *p* < 0.01; Wilcoxon rank sum test; Figure 10C). GDT impaired participants also tend to have a better ability to understand the gated speech (GDT impaired: 41.21 ± 2.5% vs. worst hearing: 47.42 ± 2.1%, *p* = 0.07; Wilcoxon rank sum test; Figure 10D). Despite the fact that the difference did not reach statistical significance due to the low number of observations, the effect size expressed as Cliff’s *d* was medium-to-large (*d* = 0.62) and therefore the difference might potentially be important. The other parameters were not significantly different (*p* > 0.05, Wilcoxon rank sum tests) (Figures 10A,E). Overall, it appears that despite that GDT is in general correlated with the speech comprehension abilities, the hearing thresholds are more important for speech understanding, at least in our group of participants.

## Discussion

In the current study we have investigated the auditory function in elderly volunteers with clinically normal hearing thresholds with the aim to identify possible central auditory dysfunctions and explore its interactions with peripheral impairments. Regular auditory tests [high frequency audiometry (HFA), speech audiometry, OAE] were complemented by additional audiometric tests (gated speech—chopper, binaural time-intensity tradeoff—laterogram, GDT, detection of clicks and short tones) and by cognitive screening (MoCA). Although the auditory thresholds of our elderly (up to 6 kHz) only show a very mild elevation due to normal aging with only minimal clinical signs of hearing pathology, our data show otherwise. Even in the elderly population without subjective hearing pathology and with only minor elevation of auditory thresholds, supracochlear structures of the auditory system show deteriorated functions. The results of the laterogram, and specifically ITD sensitivity, express altered integration of the binaural information, especially with respect of the temporal parameter. GDTs provide information about the ability of central processing of the temporal parameters of sound, which is altered independently of the function of the inner ear. Speech audiometry in babble noise and chopper, integrate the auditory information with cognitive processing and both tests show a clear deterioration with age, while the MoCA results are within the physiologic range with the exception of three borderline participants.

Pure tone audiogram represents the “gold standard” of audiometric examinations, providing basic information about the hearing ability of the subject. Under standard conditions, hearing thresholds are measured up to 8 kHz and the elevation of auditory thresholds above 25 dB HL are considered by the majority of clinicians, and also according to WHO, to be mild hearing loss. In our elderly participants, hearing thresholds up to 8 kHz showed clinically normal values, according to ISO 7029. High frequency pure tone audiometry (HFA) examines hearing thresholds up to 16 (in our case) or 20 kHz. Our elderly participants demonstrated significant deterioration of hearing above 8 kHz, which is in agreement with Matthews et al. (1997), therefore it is necessary to use HFA in specific conditions [for example: tinnitus, sensorineural hearing loss (SNHL), etc.; Wiley et al., 1998; Vielsmeier et al., 2015]. Although our study did not focus on the shape of hearing thresholds as described by Gates et al. (1990), all of the elderly participants showed a shallow sloping type. The difference in the auditory thresholds between both groups is numerically expressed by PTAV and Fowler’s percentage of hearing loss. Although the differences of these measures between elderly and young are statistically significant, both values are considered clinically normal. Furthermore, as shown by correlation analysis, the PTAV and Fowler’s coefficient are mutually dependent.

The examination of click hearing thresholds also provides a comparison of auditory thresholds, but without the frequency dependency. It also takes into account the short duration of clicks. Considering the click duration and the altered ability to track temporal changes, we expected a greater impairment of the click thresholds with aging compared to pure tone thresholds, nonetheless our results show otherwise. Although clicks are regularly used as a stimulus for brainstem audiometry, in the case of subjective audiometry the click hearing thresholds do not seem to provide any additional information to PTAV.

The analysis of the effect of stimulus duration on the auditory threshold (in our case 1 kHz tone) allows the examination of temporal processing within the inner ear and also possibly the synchrony of the auditory nerve activity (Zeng et al., 2005). The sensitivity to the stimulus duration is essential for both the individual properties of sound within the sound sequence, and for the rate characteristics of the sequence as a whole (Fitzgibbons and Gordon-Salant, 2011). As previously reported, a shorter duration of the stimulus leads to an increase of threshold (Abel, 1972), however, hearing loss does not seem to have a significant effect on duration discrimination (Fitzgibbons and Gordon-Salant, 1994) as the temporal discrimination has previously been shown as independent from the hearing threshold elevation (Grose et al., 2006; Fitzgibbons and Gordon-Salant, 2011). The deteriorated ability of duration discrimination seems to occur even in middle aged men (Grose et al., 2006). On the other hand, Fitzgibbons and Gordon-Salant (1995) showed that stimulus duration discrimination also depends on the context, suggesting that simple tone discrimination is not a good predictor for discrimination, even of the same sound presented within a more complex sequential pattern. In the case of 1 kHz tone, the hearing threshold in our elderly seems to be unaffected by aging, therefore the factor of intensity should not play a significant role in the processing of 1 kHz signal. In our study, we quantified the steepness of the dependence between the tone duration and its detection threshold using the parameters of the exponential fit. Since none of the parameters differ between young and elderly, temporal integration at the level of the inner ear appears unaffected by aging. This information is important since one of the most influential effects of aging is the decreased ability to detect fast changing (temporal) auditory cues (Grose and Mamo, 2010; Ozmeral et al., 2016a). However, comparing our results in a gap detection task and in its “inverse,” the short tone detection task, it seems that processing of the temporal aspects of sound is different in the case of detection of pauses or valleys in the stimulus envelope and in the case of the detection of amplitude peaks.

One of the explanations of impaired temporal processing that could also advocate for near normal audiograms is cochlear synaptopathy. Cochlear synaptopathy, in humans, sometimes also referred to as a hidden hearing loss, is a pathology described by Kujawa and Liberman (2009). Although initially described as a result of noise induced hearing loss, it has also recently been confirmed in aging animals as an effect of presbycusis (Sergeyenko et al., 2013). Impaired temporal processing in cochlear synaptopathy is probably due specifically to its effect on low spontaneous rate auditory nerve fibers, which would also explain the minimal elevation of auditory threshold (Liberman and Kujawa, 2017). Although the cited data seem promising, the phenomenon has not been proved to be completely transferable to humans, and the presence of cochlear synaptopathy in humans remains inconclusive (for review see Hickox et al., 2017). In our study, the peripheral auditory impairment represented by increased PTAV does not correlate with the changes of auditory temporal processing represented by ITD and GDT, which further supports the idea of independence between possible peripheral temporal processing impairment (as described in relation to cochlear synaptopathy) and more centrally located temporal processing impairments.

OAE examination provides information about the functionality of outer hair cells. As expected, in the elderly the amplitudes are lower for both types of evoked responses (TE and DP), which is a sign of hypofunction of the outer hair cell populations common in aging (Helleman et al., 2010). Lower amplitudes are accompanied by a lower number of functional outer hair cell populations (Mazelová et al., 2003; Ueberfuhr et al., 2016). The outer hair cell hypofunction in the elderly causes a deterioration of the auditory input that leads to a diminished neural signal reaching the central auditory structures. The decrease of DPOAEs amplitudes of contralateral suppression due to aging is also present in our study, although these changes were not statistically significant. This mild effect of aging on contralateral suppression suggests a relatively weak alteration of the efferent auditory system in our elderly. However, several authors have clearly demonstrated the substantial effect of aging on contralateral suppression (Kim et al., 2002; Jacobson et al., 2003). Interestingly this decrease already starts in middle age (Kim et al., 2002). The impaired function of the outer hair cells could be compensated by the proper function of the efferent auditory system. Although the exact function of the efferent auditory system remains unclear, several reports describe its positive effect on the improved extraction of auditory cues from a complex acoustic background, that could lead to an improved auditory stream segregation (Sussman et al., 2007; Füllgrabe and Moore, 2014; Pannese et al., 2015). Evidently, the deteriorated function of the efferent auditory system may contribute significantly to the problems of people with expressed presbycusis, in the identification of speech in a noisy environment.

Altered speech processing is common in the elderly. Our elderly show a normal ability to understand speech in silence (SRT), which is in agreement with the auditory threshold examination. However, the difference between young and elderly becomes apparent in SIN and seems even more profound in the understanding of temporally degraded speech, where the elderly participants need a significantly larger proportion of speech signal to understand the sentence than the young. Speech signal carries several types of information: phonetic, phonemic and syllabic and lexicosyntactics (Greenber, 1996). Although an altered auditory system could lead to the deterioration of word recognition, for example in speech (sentences) processing, the lost information (phonetic) can be compensated by use of the context (phonemic; Pichora-Fuller and Singh, 2006; Sheldon et al., 2008). However, such compensation increases the requirements on the working memory (Bopp and Verhaeghen, 2009) that might lead to an overall slowing down of the cognitive processing, especially if conditions worsen (for example in the chopper test).

One of the explanations for altered comprehension of SIN and gated speech in the elderly is their worsened ability to extract fine temporal structure information (Moore, 2016), and to track fast temporal changes (Fogerty et al., 2015). Another important factor for speech processing is sensitivity to segment duration that is significant for the better identification of stress or accent patterns (Pickett, 1999). In our case, the correlation between GDT and SIN and chopper, but absent correlation between GDT and SRT supports the idea that temporal parameters become increasingly important for speech understanding in a complex environment. Similar findings were also observed in tinnitus patients with normal hearing thresholds (Bureš et al., under review; Moon et al., 2015) that showed a beneficiary effect of sensitivity to temporal modulation on SIN in comparison to volunteers without tinnitus. Bureš et al. (under review) also showed stronger correlations between ITD and speech recognition in tinnitus patients. In this case the tinnitus plays the role of a distractor (similar speech perception in silence compared to SIN) that most likely leads to more efficient utilization of the temporal information by tinnitus patients. The detection of a gap in noise is a simple test of the temporal processing in the central auditory system, as demonstrated in experimental animals by Rybalko et al. (2010) and Suta et al. (2011) that deteriorates with age. The auditory cortex is involved in the processing of GDT since its dysfunction in rats results in prolongation of the GDT values (Syka, 2002). Age-related prolongation of GDT has also been demonstrated in humans (Mazelová et al., 2003). GDTs can be elevated, even in the elderly with normal hearing (Strouse et al., 1998). However, a certain proportion of the elderly may have GDTs similar to young subjects (Snell, 1997). In experimental animals, age-related alterations in the neuronal processing of gaps in noise were demonstrated in the inferior colliculus (Walton et al., 1998). Williamson et al. (2015) used gaps in noise auditory brainstem response (ABR) tests in a mice model of presbycusis and showed that age related changes of temporal processing already start in middle-aged animals. Age-related GDT differences become more apparent if more complex stimuli are used, such as if the gap is located near the stimulus offset or onset (He et al., 1999), or when the components bordering the gaps shift their spectral contents (Fitzgibbons and Gordon-Salant, 1994). One of the possible explanations of the deteriorated ability to track fast temporal changes is the alteration of the inhibitory mechanisms (Schatteman et al., 2008; Anderson et al., 2012). Such changes have been documented, especially in animal models of aging, as a reduction in the number of inhibitory interneurons, with lower levels of the inhibitory neurotransmitter GABA at the level of AC, as well as in the IC (Caspary et al., 2005; Ouda et al., 2015; Popelář et al., 2013), and in the case of glycine as low as in the cochlear nucleus (Frisina and Walton, 2006).

Our chopper results correspond with the results of previously used time compressed speech tests that also contain reduced linguistic and semantic cues (Gordon-Salant and Fitzgibbons, 1993), specifically naturally fast speech (Gordon-Salant et al., 2014), which alters speed processing and working memory abilities (Wingfield et al., 2006). Chopper and time-compressed speech are similar in the way that they both contain fewer contextual cues and therefore are more demanding on cognitive processing resources. Unfortunately, it is not possible to completely separate the influence of the cognitive and auditory abilities in our experimental paradigm. Although the results of MoCA screen for normal cognitive function in the elderly with the exception of three borderline participants (Nasreddine et al., 2005), the deteriorated speech signal is much more difficult to compensate by the cognitive system. Akeroyd (2008) showed that none of the commonly used cognitive tests correlate with speech in noise perception performance, supporting the idea that for auditory processing, different cognitive abilities are necessary than those examined by general cognitive tests. Decreased temporal processing also alters the integration of complex processing that uses cognitive resources and working memory (Frisina and Frisina, 1997; Grady, 2008), which could explain the lower chopper scores and normal results of the MoCA tests. Previously, an increase of working memory load has been identified as an important factor in the age related speech processing decline, especially in the case of sentences with low context cues (Wingfield, 1996). Another factor that influences the cognitive processing of speech, is the processing rate as mentioned in the case of time-compressed speech, however in the majority of individuals, a decrease of speech rate does not improve speech understanding (Gordon-Salant and Fitzgibbons, 1997). An important role that age-related changes in cognition play in the auditory ability was recently reported by Grassi and Borella (2013).

In a detailed analysis of the relationship between cognitive tasks and hearing, Füllgrabe and Moore (2014) described age related differences in the majority of cognitive tests in the young and elderly with normal hearing thresholds. Specifically, selective attention, attention switching and working memory test results were affected by aging. The results of cognitive tests were significantly correlated with altered speech processing in noise and also with temporal processing. In our study the MoCA results did not correlate with either the speech parameters or with the temporal processing parameters, suggesting that MoCA is probably not optimally suited for evaluation of the cognitive abilities related to hearing function. The effects of age and hearing loss on the processing of auditory temporal fine structure (TFS) was recently summarized by Moore (2016), taking into account that in many studies the effects of hearing loss and age have been confounded. In monaural processing, both hearing loss and aging negatively influence the output from the cochlea, represented by the TFS whereas the slowly varying envelope (ENV) is hardly affected. In the binaural version of speech stimulation, the TFS, which is important for sound localization and binaural masking level difference, is also negatively influenced by hearing loss and aging, with aging being more influential. The binaural processing of ENV also deteriorates with aging. It is difficult to compare our data with the results of psychoacoustic tests used by Füllgrabe and Moore (2014) and Moore (2016), yet even our data strongly support the view that aging is connected in humans with the deterioration of the processing of temporal aspects of the acoustical signal. For example, our data clearly show that in monaural stimulation, deterioration of the GDT frequently occurs, whereas in binaural stimulation, elderly subjects suffer from deterioration of ITD.

For some time, ABRs were used for the examination of the auditory pathway from the level of cochlear nuclei to the inferior colliculus. In our previous study (Profant et al., 2017), we showed that in spite of normal ABR results, the function of the auditory pathway might be impaired. Laterogram compares the ability to trade between the time and intensity differences of the acoustic signal coming from both ears. The ability to integrate the signal from both ears is crucial in speech processing in background noise, and in auditory stream segregation. Similarly to the decline of the peripheral and central auditory functions, orientation in acoustic space (a natural outcome of the bilateral hearing) is also altered by hearing impairment (Noble et al., 1994) and aging (Abel et al., 2000). From a clinical point of view, on the grounds of the auditory thresholds and speech perception, both groups show a normal auditory function, however there is a significant difference in the ability to extract the binaural temporal information with only minor changes in the intensity difference detection. Based on the anatomy of the auditory pathway, the integration of the acoustic cues in bilateral hearing and taking into account the normal integration of temporal information within the inner ear (duration vs. detection threshold of 1 kHz stimuli, click detection thresholds), the impaired ITD sensitivity suggests that the temporal processing impairment is probably already present at the level of auditory brainstem and inferior colliculus. The different localization of the processing of ITD vs. GDT is also supported by their very low correlation. From this perspective, the ITD detection test may be used as a complement for the gap detection test to examine the temporal processing ability at the different levels of the auditory pathway. However, it has to be taken into account that a hearing loss above 4 kHz (typical sign of presbycusis) affects ITD to a greater extent than a low frequency hearing loss (Abel and Hay, 1996). Our findings of age related decline in ITD and only minimal changes in ILD are in agreement with previous reports (Babkoff et al., 2002; Ross et al., 2007). However, when taking into account the ability to separate speech and background noise based on the different spatial localization of sources, the age related difference in ITD processing does not seem to have an effect (Füllgrabe and Moore, 2014). This is very likely due to the ability to utilize additional cues such as monaural spectral cues and ILD (Singh et al., 2008; Füllgrabe and Moore, 2014). Although the ITD as a temporal processing ability seems to play a major role in spatial orientation, another aspect affecting the temporal interaural interactions is the neural synchrony parameter, which is also negatively affected by age (Ozmeral et al., 2016b). For the binaural interactions and specifically spatial coding, the neural synchrony at the level of medial superior olive is important and is negatively affected by insufficient inhibitory inputs due to aging (Pecka et al., 2008).

One of the aims of our project was to better characterize SNHL. According to the reported data it seems that even in a homogenous population of elderly with clinically normal hearing (deterioration of auditory thresholds is in agreement with their age “hearing age”), clear differences are present. There is variability at the level of the inner ear, auditory pathway and also at the level of auditory cortex and in interactions with the cognitive network. For the purpose of improved identification of SNHL, we correlated a basic inner ear parameter—PTAV—with a basic central hearing parameter—GDT. The auditory periphery/center correlation proposes at least four possible subgroups: the real “golden ears” with almost perfect function of the auditory periphery and center, groups with either good periphery and declined central auditory function or declined peripheral auditory function and good central function, and a fourth group with both functions deteriorated. Another finding is that in our elderly the relatively minor degree of purely central pathology represented by higher GDT (GDT impaired subgroup of elderly) has only limited influence on speech processing, especially in comparison with the effect of peripheral function represented by PTAV. Additional comprehensive examination is needed, especially in the elderly with a more profound hearing deterioration, to further stress this classification and potentially add more presbycustic subpopulations. Our approach might complete the missing pieces in several theories of presbycusis as proposed by CHABA (1998).

Our data present a new insight (specifically the chopper and laterogram tests) into auditory processing in humans with a potential impact on the diagnosis of SNHL. This is especially in contrast with the classic clinical approach to the diagnosis of hearing loss that uses only limited examination. As presented above, none of our elderly participants had an auditory function that would be considered by clinical standards as pathological, however our detailed tests show otherwise. We believe that in specific diagnosis such as presbycusis or potentially tinnitus, a more thorough examination is necessary. We aim to use our battery of tests to further classify the different subtypes of presbycusis that might eventually lead to a different approach of how to rehabilitate hearing impairment. Nonetheless, at this point there is no clear clinical recommendation for the use of a novel part of the test battery since more data is needed for validation. Among the clinically available tests in specific cases when a patient’s subjective feeling does not correlate with the results of basic audiometric examination, HFA, SIN and the effect of contralateral suppression on OAE should be complemented.

Although our results provide a new insight into the pathophysiology of the age related changes in the auditory system, it is necessary to point out that the novel tests (specifically laterogram, chopper and short tone audiogram) have not been validated yet. However, based on the results of these tests and their correlation with the standardized auditory and cognitive measurements, convergent validity approach can be implemented. Nonetheless, in the near future, we plan to produce validated results of these tests for each age group (in 10 year intervals) that would provide reference data and allow a more general use of these tests.

## Conclusion

Presbycusis is a complex disorder affecting different parts of the auditory system. Although the elevation of hearing thresholds is its most common expression indicating inner ear (peripheral) pathology, several other central auditory features are affected as well. The presented data demonstrate the deterioration of the interaural auditory processing (laterogram), specifically of temporal information (ITD), decreased ability of auditory cortex to identify fast signal changes (GDT) and an overall decline of the integration of auditory and cognitive abilities (speech in noise, chopper). The analysis of the interactions of peripheral and central auditory abilities showed a stronger influence of peripheral function (expressed as hearing thresholds) than temporal processing ability on speech perception in silence, however in a more natural environment such as with the addition of noise to speech, the role of temporal processing increases rapidly. Our data reveal several pathologies causing presbycusis and suggest that they are to some extent independent of each other and, in particular, that hearing threshold elevation does not predict the state of the supracochlear auditory function.

## Data Availability

All datasets generated for this study are included in the manuscript.

## Author Contributions

OP: manuscript preparation, examination, auditory tests creation, statistical analysis, work load ca 25%. MJ: examination, software preparation, auditory tests creation, work load ca 15%. ZB: software preparation, auditory tests creation, statistical statistical analysis, manuscript preparation, work load ca 15%. VV: examination, software preparation, work load ca 10%. DK and VS: examination, database administration, work load ca 10%. JK: software preparation, work load ca 5%. JS: manuscript preparation, auditory test creation, project overview, work load 10%.

## Conflict of Interest Statement

The authors declare that the research was conducted in the absence of any commercial or financial relationships that could be construed as a potential conflict of interest.

## Abbreviations

ABR: auditory brainstem responses
DP: distortion product OAE
“elderly”: elderly group
GDT: gap detection threshold
HFA: high frequency audiometry
ILD: interaural level difference
ITD: interaural time difference
MoCA: Montreal cognitive assessment
OAE: otoacoustic emissions
PTAV: pure tone average
SIN: speech in noise
SNHL: sensorineural hearing loss
SRT: speech reception threshold
TE: transient evoked OAE
“young”: young controls.
